# Death’s Summer Campaign

**Published:** 1876-06

**Authors:** 


*TWENTY-THIRD YEAR OF*

### HALL'S JOURNAL OF HEALTH.

***E. H. Gibbs, M.D., Editor.***

VOL. XXIII.] JUNE, 1876. [No. 6.

### *DEATH'S SUMMER CAMPAIGN.*

IN regard to those diseases of summer that carry off thousands of children, it may be said that "history repeats itself." Especially is it true in this country, no portion of which is exempt from the ravages of the destroyer. During the months of July, August, and September, death reaps its harvest. In some localities the fatality is such as to cause the greatest alarm. People ask if nothing can be done to arrest the disease; if some better plan of treatment can not be resorted to than giving opium in the form of "Soothing Syrup," or some other abominable compound. We venture the opinion that two children are destroyed by this murderous treatment where one dies of the disease. In other words, two children would recover without medical treatment, where one now recovers in spite of opium; and our advice is, never to give opium, in any form or in the smallest quantity, to a child under fifteen years, for any disease whatever. A casual glance at the causes and pathology of the disease, will suggest its proper treatment.

Teething is not the direct cause of "Cholera Infantum" as many suppose. The pain produced by difficult dentition prevents the child from taking proper nourishment, rest, or sleep. The system becomes debilitated, and is prepared to take on a diseased action. The first manifestations of disease are copious, watery discharges from the bowels. The parents become alarmed, and dose the child with paregoric, "Soothing Syrup," or some other preparation of opium. This treatment will often check the discharges from the bowels within six hours, and destroy the poor baby in about forty-eight hours. Death *naturally* results from this treatment. The fluid that collects in the stomach and bowels is not allowed to escape by the natural channels, which have been blocked by the opium. It is constantly accumulating, and when the stomach and intestines become gorged, a portion forces its way into the lungs and brain, and the child dies of congestion, and this is always the cause of death; still intelligent men—many of them physicians, we are sorry to say—fold their arms and piously talk about the dispensations of Providence. What blasphemy! No child with a good constitution will die of "Cholera Infantum" if properly treated and nursed. The treatment should be directed to the liver exclusively, as that is the seat of all the trouble. When the medicine has had time to act on the liver, the condition of the bowels will rapidly improve; but the discharge should never be checked in any other way.

